# Facile and eco-friendly fabrication of biocompatible hydrogel containing CuS@Ser NPs with mechanical flexibility and photothermal antibacterial activity to promote infected wound healing

**DOI:** 10.1186/s12951-023-02035-6

**Published:** 2023-08-10

**Authors:** Ye Guo, Bingqing Xie, Min Jiang, Lingling Yuan, Xueyu Jiang, Silei Li, Rui Cai, Junliang Chen, Xia Jiang, Yun He, Gang Tao

**Affiliations:** 1 Oral and Maxillofacial Reconstruction and Regeneration of Luzhou Key Laboratory, The Affiliated Stomatological Hospital of Southwest Medical University, Luzhou, 646000 China; 2 Institute of Stomatology, Southwest Medical University, Luzhou, 646000 China; 3 Department of Oral and Maxillofacial Surgery, The Affiliated Stomatological Hospital of Southwest Medical University, Luzhou, 646000 China; 4 Department of Oral and Maxillofacial Surgery, The Affiliated Hospital of Southwest Medical University, Luzhou, 646000 China

**Keywords:** Copper sulfide nanoparticles, Hybrid hydrogel, Mechanical flexibility, Photothermal therapy, Wound healing

## Abstract

Bacterial infections can significantly impede wound healing and pose a serious threat to the patient’s life. The excessive use of antibiotics to combat bacterial infections has led to the emergence of multi-drug-resistant bacteria. Therefore, there is a pressing need for alternative approaches, such as photothermal therapy (PTT), to address this issue. In this study, for the first time, CuS NPs with photothermal properties were synthesized using sericin as a biological template, named CuS@Ser NPs. This method is simple, green, and does not produce toxic and harmful by-products. These nanoparticles were incorporated into a mixture (XK) of xanthan gum and konjac glucomannan (KGM) to obtain XK/CuS NPs composite hydrogel, which could overcome the limitations of current wound dressings. The composite hydrogel exhibited excellent mechanical flexibility, photothermal response, and biocompatibility. It also demonstrated potent antibacterial properties against both Gram-positive and negative bacteria via antibacterial experiments and accelerated wound healing in animal models. Additionally, it is proved that the hydrogel promoted tissue regeneration by stimulating collagen deposition, angiogenesis, and reducing inflammation. In summary, the XK/CuS NPs composite hydrogel presents a promising alternative for the clinical management of infected wounds, offering a new approach to promote infected wound healing.

**Supplementary Information:**

The online version contains supplementary material available at 10.1186/s12951-023-02035-6.

## Introduction

The skin is the body's first line of defense against microbial invasion. When the integrity of the skin is compromised, it can lead to serious infections [1]. Bacterial infections of skin burns, wounds, and ulcers have become a serious threat to human health, leading to septicemia and tissue necrosis [2]. Persistent infections can delay wound healing and require amputation in the most severe cases, which can cause physical and emotional pain for patients [3]. Traditional wound dressings, such as bandages and gauze, lack the antimicrobial properties to treat infected wounds effectively [4]. Although antibiotics are usually combined with wound dressings to inhibit infection, their improper use can induce multi-drug resistant bacteria that are difficult to eliminate, causing more severe local or even systemic infections [5]. Therefore, there is an urgent need to develop novel wound dressings combined with effective antimicrobial strategies to treat bacterial infections.

Photothermal therapy (PTT) has emerged as a potential therapy in recent years, utilizing photothermal agents (PTA) to convert light energy into heat energy and eliminate bacterial infections in wounds without inducing bacterial drug resistance [6, 7]. PTA plays a crucial role in PTT, and it is required to have high photothermal conversion efficiency, photostability, and low biotoxicity [8]. Copper sulfide nanoparticles (CuS NPs) are a type of semiconductor PTA that exhibit high photothermal conversion, good biocompatibility, and low cost [9, 10]. Conventional methods for the preparation of CuS NPs include hydrothermal [11], precipitation [12], emulsion [13], and microwave heating methods [14]. However, these conventional methods often involve harsh reaction conditions and generate environmentally polluting by-products. The preparation of inorganic nanomaterials by in situ synthesis has the advantage of low environmental pollution and has attracted great interest in recent years [15, 16]. The in situ synthesis of CuS NPs using biomolecules as biological templates is a novel alternative [17].

Sericin (Ser) is a natural macromolecular viscous protein wrapped in the surface layer of silk fibroin and is one of the two main proteins that make up the cocoon of silkworms. Sericin has cell adhesion properties, good biocompatibility, and low immunogenicity, which has been used in combination with other polymers to form wound dressings in different forms [18, 19]. It has great potential in wound dressing and skin repair, with beneficial effects on fibroblasts due to its various bioactivities. On the other hand, the sericin molecule has a loose and disordered spatial structure and contains several amino acid residues with long side chains and polar hydrophilic groups on the surface of the polypeptide chain, which can coordinate with copper ions [20]. Previous studies have successfully used sericin to synthesize inorganic nanomaterials in situ, including silver nanoparticles and hydroxyapatite [18, 21, 22]. Therefore, we believe that sericin has a small enough molecular gap to control the particle size of CuS to form nanoparticles in situ, which can be used as a potential biomolecular template for the preparation of CuS nanoparticles.

Hydrogels have been more widely used as drug carriers in biomedical applications [23], with good biocompatibility and biodegradability as well as 3D network structure like extracellular matrix (ECM) [24, 25]. Currently, many studies have developed hydrogels with different characteristics that promote wound healing in different environments. Chen et al. reported a hydrogel based on collagen and starch, which shows excellent adhesion and hemostatic properties [26]. Hu et al. used poly(vinyl alcohol) (PVA), 3,4-dihydroxyphenylalanine (DOPA), and Cu^2+^ to form a PVA-DOPA-Cu (PDPC) hydrogel, which has antioxidative and antibacterial properties [27]. Compared to conventional dressings, hydrogels provide a good healing environment and prevent microbial invasion [28]. In addition, hydrogels can absorb wound exudate while maintaining a moist environment without causing secondary trauma due to adhesion to the wound [29]. Xanthan gum and konjac glucomannan cannot form a gel when dispersed in water alone but can produce a strong synergistic effect when they are mixed [30]. Briefly, xanthan gum has a double helix structure at room temperature, which uncoils at high temperatures and intertwines with konjac glucomannan (KGM), then recovers the double helix and forms a network structure with KGM after cooling. The presence of a cross-linked network forms the hydrogel of xanthan gum/konjac glucomannan (XK), which has good mechanical properties and flexibility and can cope with various environments and unexpected conditions such as violent impact and extrusion [31]. The healing of the wound can be accelerated with adequate protection of the wound by the hydrogel. However, the XK hydrogel cannot resist bacteria, so we propose to prepare XK hydrogels loaded with CuS NPs that have the functions of wound protection, photothermal antibacterial activity, and promotion of wound healing.

**Fig. 1:**
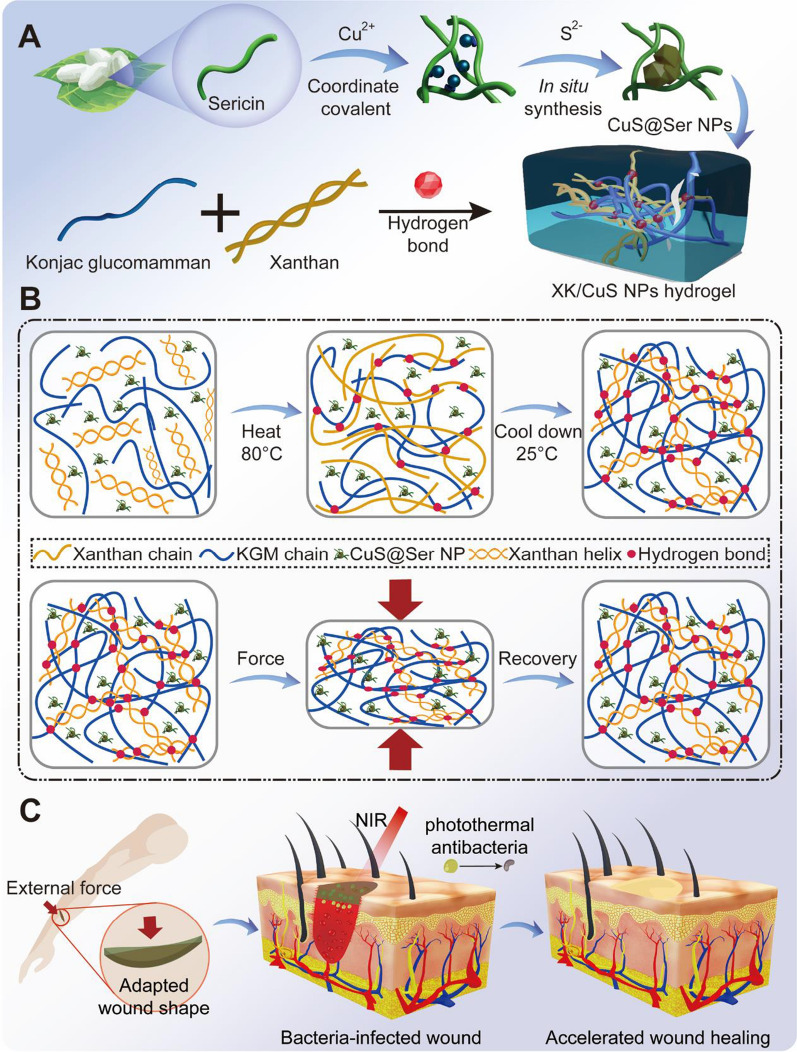
**A** The in situ synthesis of CuS@Ser NPs and synthesis of XK/CuS NPs hydrogel. **B** Schematic illustration of the structure and mechanical flexibility of XK/CuS NPs hydrogel. **C** XK/CuS NPs hydrogel can protect the wound from external force and are capable of photothermal antibacterial, thus accelerating wound healing

In this study, CuS NPs with photothermal properties were successfully synthesized in situ by a simple and green method using sericin as a biological template (Fig. 1A). On this basis, we prepared XK/CuS NPs hydrogels by introducing xanthan glucomannan and konjac gum complexes into the CuS NPs solution (Fig. 1B). Further, systematically characterized the microstructure and mechanical properties of XK/CuS NPs hydrogel, then investigated the antibacterial activity of the hydrogel against *Escherichia coli* (*E. coli*) and *Staphylococcus aureus* (*S. aureus*). The hydrogel exhibited excellent compression stability, mechanical flexibility, and photothermal antibacterial ability. Furthermore, we conducted biocompatibility tests using human umbilical vein endothelial cells (HUVECs) and L929 cells (mouse fibroblasts). In addition, wound healing-promoting effects were also analyzed in vivo. This study demonstrates that XK/CuS NPs hydrogel can produce photothermal effects to inhibit bacterial proliferation and can promote the healing of infected skin wounds, which has promising applications (Fig. 1C).

## Experimental

### Materials

Silkworm cocoons were obtained from the Seri Cultural Research Institute (Jiangsu, China). Copper sulfate pentahydrate (CuSO_4_·5H_2_O), ammonium hydroxide aqueous solution (NH_3_·H_2_O), sodium sulfide nonahydrate (Na_2_S·9H_2_O), xanthan gum, and KGM were obtained from Aladdin (Shanghai, China). All other reagents, including Dulbecco's modified eagle medium (DMEM; Gibco, CA, USA), Gibco Roswell Park Memorial Institute (RPMI) 1640 medium (Gibco, CA, USA), Fetal Bovine Serum (Gibco, CA, USA), 4% paraformaldehyde, 1% penicillin–streptomycin (Beyotime, Beijing, China), 0.25% Trypsin–EDTA (Gibco, CA, USA), Live/Dead^®^ viability (Thermo Fisher Scientific, MA, USA), cell counting kit (APE-BIO, TX, USA), FITC Phalloidin (Solarbio, Beijing, China), were used directly.

### Preparation of sericin solution

Sericin solution was prepared as the previous method [18]. In brief, silkworm cocoons were cut up and put in deionized water, boiled under 0.1 MPa at 121 °C for 20 min, and filtered to remove the silk fibroin fibers to obtain sericin solution. The concentration is adjusted to 1% (w/v) for use.

### Synthesis of CuS@Ser NPs

To prevent excessive local Cu^2+^ concentration, 2 mL of 40 mM CuSO_4_ was mixed with 7 M NH_3_·H_2_O (100 μL) to obtain [Cu(NH_3_)_4_]^2+^ solution. Afterward, it was added to 1% sericin solution (10 mL) with stirring, then 4 M Na_2_S solution was added dropwise into the mixture until the color of the solution did not change. The volume was added to 40 mL with deionized water to obtain a 2 mM CuS@Ser NPs solution named CuS2. In addition, CuS1 and CuS0.5 were obtained in the same way. All reactions were conducted at indoor temperature.

### Characterization of CuS@Ser NPs

The morphology and size of CuS@Ser NPs were analyzed using a transmission electron microscope (TEM, JEM-2100, Tokyo, Japan). The components and distribution of the elements were measured by an X-ray energy-dispersive spectroscopy (EDS) detector. Ultraviolet–visible-near-infrared spectra (UV–vis-NIR) of samples were obtained by UV–vis spectrophotometer (TU-1810, Shanghai, China). Fourier transform infrared (FTIR) spectra of CuS, sericin, and CuS@Ser NPs were measured by FTIR spectrometer (WQF-530, Beijing, China). The chemical state was determined by X-ray photoelectron spectroscopy (XPS, Shimadzu Kratos AXIS Ultra DLD, Nagoya, Japan).

### Photothermal property of CuS@Ser NPs

To test the photothermal property of CuS@Ser NPs solution, irradiating different concentrations of CuS@Ser NPs solution with various light intensities (0.5, 1.0, and 1.5 W/cm^2^) under 808 nm NIR laser. Briefly, the CuS@Ser NPs solution was set in a 1.5 mL centrifuge tube and exposed to an 808 nm laser. The temperature changes and corresponding thermograms were recorded in real-time with an infrared thermographic camera (FLIR, E8-XT, Wilsonville, OR, USA). Afterwards, irradiating CuS@Ser NPs solution with 808 nm laser (1.5 W/cm^2^) for 5 min, switching off and cooling down to room temperature, as such cycle three times to test the photothermal stability. The temperature changes were also recorded with the infrared thermographic camera.

### Preparation of XK/CuS NPs hydrogel

Briefly, KGM was added to deionized water (20 mL), heated to 80 °C, and stirred uniformly with a high-speed homogenizer; then, xanthan gum was added and stirred uniformly. Adjust the pH of the solution to 7.0, and the mixture was transferred to clean molds and cooled to an indoor temperature to obtain XK hydrogel. Of which the mass fraction of KGM and xanthan gum was 1%, and the mass ratio was 6:4. The deionized water was replaced with CuS0.5, CuS1, and CuS2 solutions to obtain XK/CuS NPs hydrogels, named XK/CuS0.5, XK/CuS1, and XK/CuS2, respectively.

### Characterization and physical properties of XK/CuS NPs hydrogel

The hydrogel samples were lyophilized in a freeze-dryer, and then the microstructures of the samples were analyzed by field emission scanning electron microscopy (FE-SEM, LEO435VP, Cambridge, UK), and the distribution of various elements was obtained by EDS images. FTIR spectra of XK and XK/CuS hydrogels were measured by FTIR spectrometer (WQF-530, Beijing, China). Compression tests and cyclic compression experiments were performed on XK, XK/CuS0.5, XK/CuS1, and XK/CuS2 hydrogels using a universal mechanical tester (INSTRON, 5965, Massachusetts, USA). Briefly, samples with a height of 15 mm were compressed to rupture at a rate of 5 mm/min, and stress–strain curves were recorded. Next, each sample was compressed to 60% strain, then restored to the free state, and cycled 30 times to record the stress–strain curves. To test the stability of the hydrogels, the same mass of XK, XK/CuS0.5, XK/CuS1, and XK/CuS2 hydrogels were soaked in PBS, photographed daily to confirm the status, and weighed after drying.

### Biocompatibility test of XK/CuS NPs hydrogel

To test the biocompatibility of the hydrogel, CCK-8 assay, Live/Dead staining, and cytoskeleton staining were used. 10 g of hydrogels (XK, XK/CuS0.5, XK/CuS1, XK/CuS2) were soaked in 40 mL of DMEM or RPMI 1640 medium containing 10% FBS and 1% penicillin–streptomycin for 24 h to acquire the leachates, respectively. HUVECs and L929 cells were cultured for 24 h in DMEM or RPMI 1640 medium containing 10% FBS and 1% penicillin–streptomycin, respectively. And then, replaced the complete medium with the leaching solutions, and the culture was continued for the 1st, 3rd, and 5th days and performed following assays. CCK-8 assay kit was used to measure cell viability. Briefly, add 10 µL of CCK-8 reagent to the medium (100 µL), and the cells were co-cultured for 1.5 h at 37 °C. The OD value was recorded at 450 nm by a microplate reader (TECAN Infinite M200PRO, China). Further, HUVECs and L929 cells were cultured with leachates for the 1st, 3rd, and 5th days, respectively. Then, incubate with live/dead staining solution for 0.5 h and observe under a fluorescent microscope (Leica, DMi8, Germany). In addition, cells cultured with leachates for 3 days were fixed with 4% paraformaldehyde for 10 min and treated with 0.5% Triton X-100 for 5 min. FITC Phalloidin solution was used to stain the cytoskeletal actin for 0.5 h. After washing with PBS 3 times, the nuclei were stained with DAPI for 30 s, observed by fluorescence microscope.

### Photothermal property of XK/CuS NPs hydrogel

To test the photothermal property of XK/CuS NPs hydrogel, irradiating different hydrogels (XK, XK/CuS0.5, XK/CuS1, XK/CuS2) with different light intensities (0.5, 1.0, and 1.5 W/cm^2^) under 808 nm laser, respectively. Briefly, the hydrogel was set in a dish and irradiated with an 808 nm laser. The temperature changes and corresponding thermograms were measured in real-time with the infrared thermographic camera. Afterward, irradiate XK/CuS1 hydrogel with the laser (1.5 W/cm^2^) for 5 min, switch it off, then cool down to room temperature; cycle three times to test the photothermal stability. The temperature changes were also recorded with the infrared thermographic camera.

### Antibacterial assay in vitro

PBS solution (blank control), XK hydrogel, and XK/CuS1 hydrogel were placed in a 6-well plate. 100 μL of *S. aureus* and *E. coli* suspensions (1 × 10^7^ CFU/mL) were added to the surface of the hydrogels. And then, the hydrogels were irradiated with an 808 nm NIR laser for 5 min. PBS solution (2 mL) was added to each well and shaken for 10 min. Collecting the bacterial suspension (100 μL) and coated on LB solid medium and incubated at 37 °C for 12 h, then counted colonies. Moreover, bacterial live/dead staining was performed. Bacterial precipitates were obtained by centrifugation of the bacterial suspension at 12,000 rpm for 10 min, then incubated with a Live/Dead bacterial imaging kit for 15 min at room temperature in the dark and observed under the fluorescent microscope.

### Wound healing assay in vivo

The six-week-old male SD rats were acquired from the animal experiment center of Southwest Medical University. A round full-skin wound of 10 mm in diameter was punched on the back of each SD rat with a hole punch. All animal experiments are performed in accordance with the regulations of the animal ethics committee of Southwest Medical University. 100 μL of *S. aureus* suspension (1 × 10^7^ CFU/mL) was injected into each wound [32]. XK and XK/CuS1 were added to the wounds, respectively, and the control group was left untreated and irradiated with 1.5 W NIR light for 5 min. Each wound was renewed in the dressing and photographed on the 0th, 3rd, 6th, 9th, and 12th day, respectively. The wound area was analyzed with Image J.

### Histological and immunohistochemical analysis

Rats were sacrificed on the 6th and 12th day, and surrounding wound tissue was obtained, fixed in 4% paraformaldehyde, paraffin-embedded, and sliced. Hematoxylin and eosin (H&E) staining and Masson trichrome staining was conducted on the tissue sections, respectively. In addition, the sections were incubated with anti-rabbit CD31 and anti-rabbit TNF-α (Servicebio, China) at 4 °C for 24 h, then incubated with HRP-labeled goat anti-rabbit IgG (H + L) at indoor temperature for 2 h. Finally, they were imaged by a digital pathology slide scanner (KF-PRO-002, China) and observed by K-Viewer (1.7.0.23) X64.

### Statistical analysis

All experiments were repeated three times, and the results were expressed as mean ± standard deviation. Statistical differences were analyzed by employing the One-way ANOVA by soft SPSS 26.0. p < 0.05 indicated a significant difference.

## Results and discussions

### Synthesis and characterization of CuS@Ser NPs

**Fig. 2:**
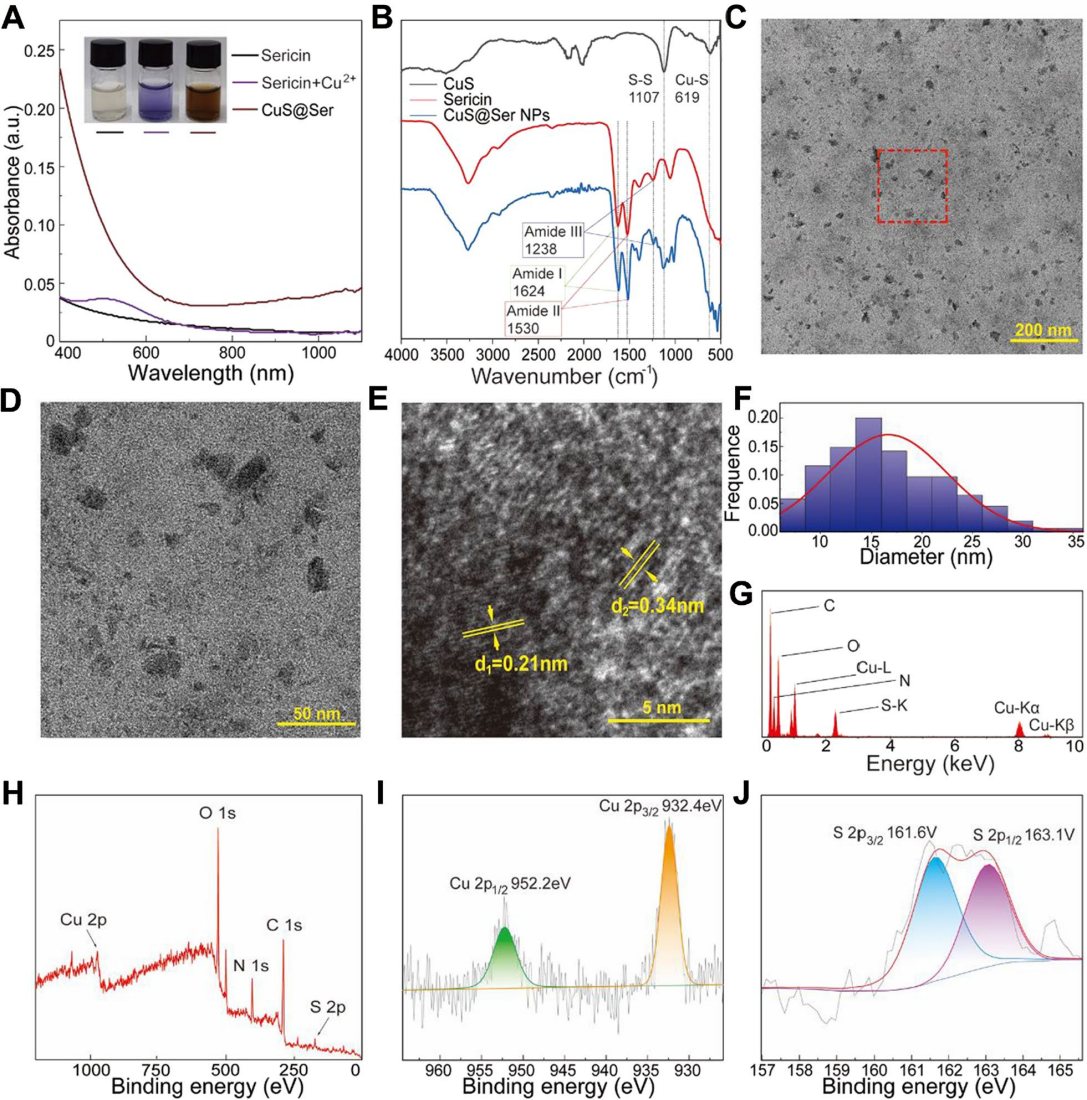
Characterizations of the CuS@Ser NPs. **A** UV–vis absorption spectra of sericin, sericin + Cu^2+^, and CuS@Ser NPs solution. **B** FTIR spectra of the CuS, sericin, and CuS@Ser NPs solution. **C**, **D** TEM images,** E** HRTEM image, **F** particle size distribution, and **G** EDS of CuS@Ser NPs.** H** XPS wide-range spectra of CuS@Ser NPs. High-resolution spectra of **I** Cu 2p and **J** S 2p in CuS@Ser NPs

CuS NPs were synthesized based on sericin as a biological template and characterized by UV–vis spectrophotometer, FTIR, TEM, EDS, and XPS. As shown in Fig. 2A, the sericin solution turned purple after the addition of [Cu(NH_3_)_4_]^2+^ and brown after the addition of Na_2_S solution without precipitation, which was attributed to the abundant hydroxyl groups in the sericin molecules complexed with Cu^2+^. After adding S^2−^, the nano-scale molecular gap caused by the irregular curl structure of sericin prevented CuS from over-aggregating, thus forming CuS NPs with uniform particle size and wrapped by the sericin molecules. The UV–vis spectra of the brown solution (CuS@Ser) showed obvious absorption in the section of 800–1100 nm, suggesting that it can absorb NIR light, consistent with previous study reports [33]. The FTIR spectra of CuS, sericin, and CuS@Ser NPs are exhibited in Fig. 2B. The FTIR spectrum of CuS exhibits peaks at 619 cm^−1^ and 1107 cm^−1^, attributed to the stretching vibrations of Cu–S and S–S [34, 35]. In the spectrum of sericin, there are strong bands at 1624, 1530, and 1238 cm^−1^, which correspond to the characteristic peak of amide I, II, and III, respectively [36]. The spectrum of CuS@Ser NPs has the characteristic peaks of Cu–S, S–S, and amide I, II, and III, suggesting that CuS NPs were successfully synthesized in sericin.

The microscopic morphology of CuS@Ser NPs was analyzed by TEM, as shown in Fig. 2C–E. The particle size of CuS NPs ranged from 10 to 25 mm, which were uniformly dispersed in the sericin solution without excessive aggregation (Fig. 2C, D, F). HRTEM showed clear lattice stripes (Fig. 2E), which were 0.34 nm and 0.21 nm, corresponding to the (101) and (110) crystal faces [37, 38]. Further analysis of the samples by EDS and XPS demonstrated the presence of Cu and S (Fig. 2G, H). The high-resolution XPS spectra exhibited that the binding energy peaks at 952.2 and 932.4 eV coincide with Cu 2p_1/2_ and Cu 2p_3/2_, respectively (Fig. 2I). As seen in Fig. 2J, the peaks are at 161.6 and 163.1 eV, belonging to S 2p_3/2_ and S 2p_1/2_, respectively [39–41]. These results confirm the successful synthesis of CuS NPs together.

### Photothermal property of CuS@Ser NPs

**Fig. 3:**
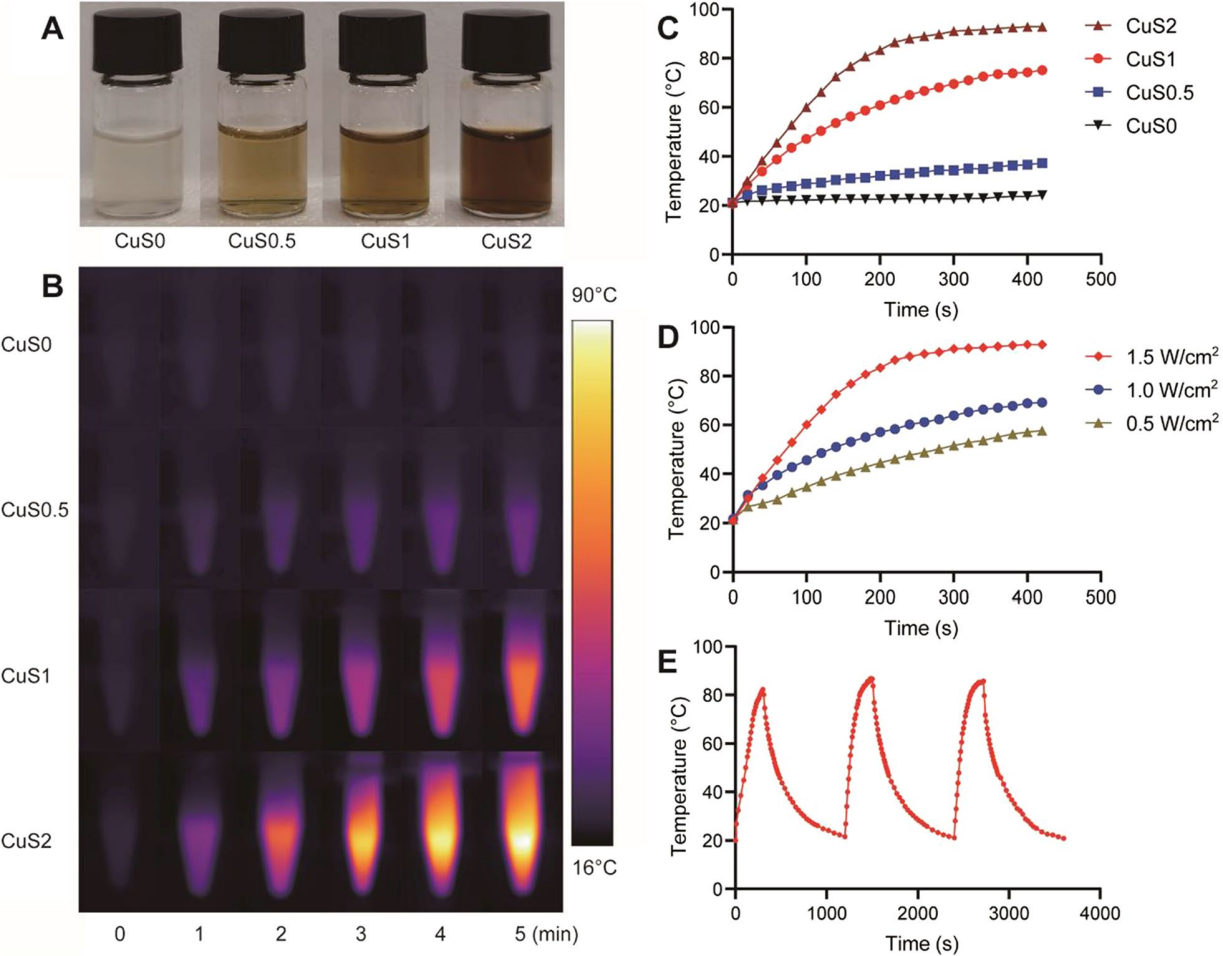
Photothermal property of CuS@Ser NPs solutions. **A** Photograph of different concentrations of CuS@Ser NPs solutions. **B**, **C** Infrared images and temperature profiles of CuS@Ser NPs solutions under 808 nm laser (1.5 W/cm^2^). **D** Temperature profiles of CuS2 under a laser power density of 0.5 W/cm^2^, 1.0 W/cm^2^, and 1.5 W/cm^2^. **E** Temperature profiles of CuS2 for 3 on–off cycles under 808 nm laser (1.5 W/cm^2^)

We tested the photothermal properties of different concentrations of CuS@Ser NPs solutions under NIR light irradiation at 808 nm. With the increase of CuS content, the color of the solution gradually deepened (Fig. 3A), while the photothermal properties also gradually enhanced (Fig. 3B). The temperature of CuS@Ser NPs also increased as the irradiation time became longer. Figure 3C displays the temperature changes of CuS@Ser NPs with various concentrations under a 1.5 W/cm^2^ 808 nm laser. Due to the lack of CuS NPs, the CuS0 solution showed no temperature rise under laser irradiation. In contrast, the temperatures of CuS0.5, CuS1, and CuS2 solution increased to 37.3 °C, 75.1 °C, and 97.9 °C, respectively, and reached stability after 300 s. Figure 3D shows the temperature of CuS2 solution change under different power of 808 nm NIR irradiation. With the increase of laser power, the maximum temperature of the CuS2 solution also rises, and the temperature stabilization was most rapidly reached at 1.5 W. It helps to precisely regulate the maximum temperature to ensure the stable performance of sterilization effects [42]. Then the on–off cycling experiment was carried out. As shown in Fig. 3E, the solution could still rise to the previous temperature after three on–off cycles, indicating that CuS@Ser NPs have good photothermal stability and can be used repeatedly, which is consistent with the CuS NPs synthesized by BSA in previous work [43].

### Characterization and physical properties of XK/CuS NPs hydrogel

**Fig. 4:**
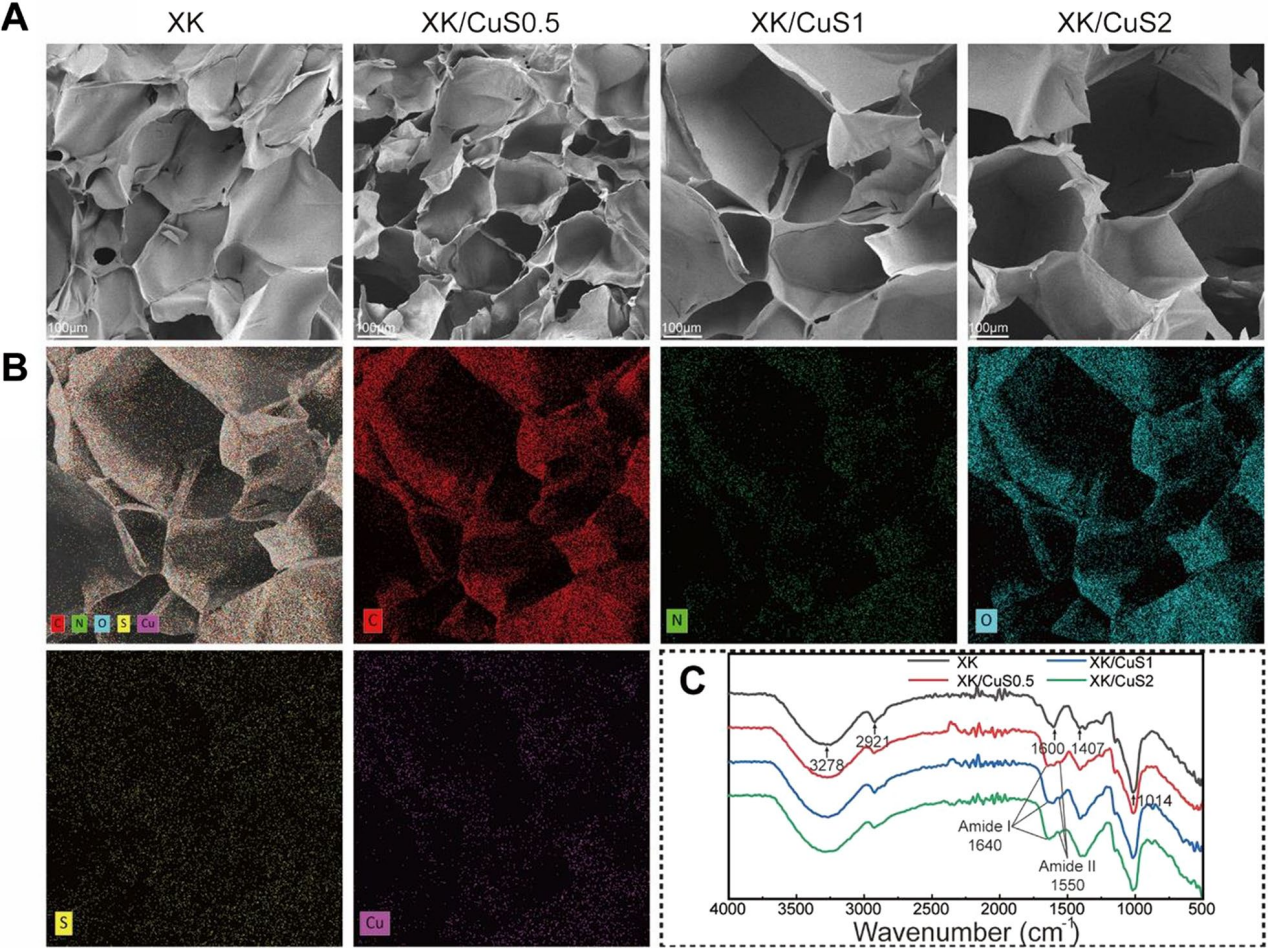
Microscopic morphology and FTIR spectra of XK and XK/CuS hydrogels. **A** SEM images of XK, XK/CuS0.5, XK/CuS1, and XK/CuS2 hydrogels. **B** EDS elemental mapping of XK/CuS1 hydrogel. **C** FTIR spectra of XK and XK/CuS hydrogels

Polysaccharides have been widely used in tissue engineering materials because of their biocompatible characteristics [44]. As excellent polysaccharides, xanthan gums were mixed with KGM to synthesize XK hydrogel and loaded with CuS NPs. The microscopic morphologies of XK, XK/CuS0.5, XK/CuS1, and XK/CuS2 were analyzed by SEM, as shown in Fig. 4A. The main body of XK and XK/CuS hydrogels has a three-dimensional mesh structure with many pores, which has the advantages of nutrients and oxygen exchange [45]. The addition of CuS NPs did not affect the porous structure. In addition, the elemental distribution of XK/CuS1 hydrogel was analyzed by EDS (Fig. 4B). The results showed that Cu and S are uniformly distributed in the hydrogel structure, which proves that CuS NPs were successfully loaded and uniformly dispersed in the hydrogel. Moreover, FTIR spectra of XK and XK/CuS hydrogels were given in Fig. 4C. The FTIR spectrum of XK hydrogel shows characteristic peaks at 3278, 2921, 1600, 1407, and 1014 cm^−1^, attributed to –OH stretching, C–H stretching, –OH bending, –CH_3_, and C–O–C symmetrical stretching, respectively [46]. The FTIR spectra of XK/CuS0.5, XK/CuS1, and XK/CuS2 all show characteristic peaks of XK hydrogel, also show a characteristic peak at 1640 cm^−1^ attributed to amide I group of sericin, and the peak at 1550 cm^−1^ attributed to the amide II group of sericin [36]. While the FTIR spectrum of XK doesn’t have characteristic peaks of the amide II group and amide I group. This result indicated that the CuS@Ser NPs were successfully incorporated into the XK hydrogel.

**Fig. 5:**
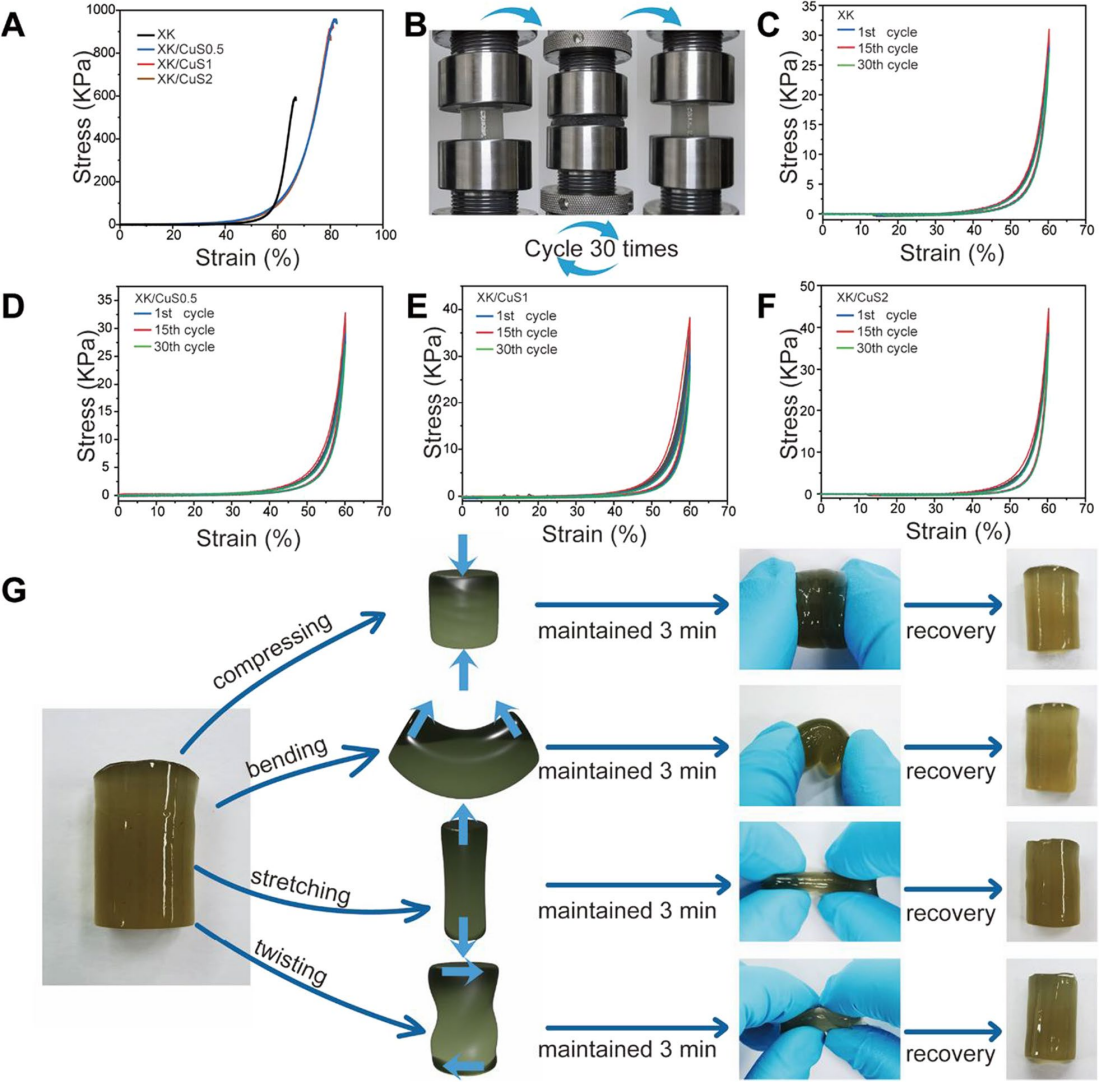
Mechanical performance of XK and XK/CuS hydrogels. **A** The compression stress–strain curves of XK and XK/CuS hydrogels. **B** Schematic diagram of cyclic compression assay. **C**–**F** The cyclic compression stress–strain curves of XK and XK/CuS hydrogels. **G** Deformation and recovery of XK/CuS1 hydrogels under the action of different forces

Wound dressings with good mechanical properties can protect the wound against unexpected situations such as external impact [47]. Mechanical flexibility can make it suitable for various environments [48]. As a wound dressing, the hydrogel needs to have sufficient mechanical properties to resist breakage caused by external factors such as friction [49]. The mechanical properties of single xanthan gum and konjac glucomannan are too weak to form a hydrogel. In contrast, the binding of the two components can form a hydrogel with good mechanical strength as well as flexibility [29]. The compressive strength of XK and XK/CuS hydrogels was evaluated by compression test. As shown in Fig. 5A, the XK hydrogel fractured at 65% strain when the compressive stress reached 600 kPa. In contrast, the mechanical properties of XK/CuS hydrogels are more excellent, with maximum compressive strains up to 80%, while the compressive stress exceeds 900 kPa. The increased mechanical properties of XK/CuS hydrogels are attributed to the nanoparticle-polymer interactions between the two polysaccharides and CuS NPs. When the hydrogel deforms, the stress concentration creates cavities around the CuS NPs, resulting in the dissipation of a large amount of energy and enhancement of the hydrogel’s mechanical properties [50–53].

The compressive stability of XK/CuS hydrogels was evaluated by cyclic compression experiments, which were cycled 30 times at 60% compressive strain, and Fig. 5B shows the scheme of this experiment. All hydrogels recovered their initial shapes after 30 cycles, implying that they have good compression stability (Fig. 5C–F). Moreover, different types of forces were applied to the XK/CuS NPs hydrogels (compressing, bending, stretching, twisting), which were maintained for 3 min and then released, and all hydrogels returned to their original state (Fig. 5G). The above results indicate that the XK/CuS NPs hydrogel has mechanical flexibility. In summary, XK/CuS hydrogels have excellent mechanical properties and can cope with most of the tissue defects that require barriers.

The stability of hydrogels was evaluated by degradation experiments. The same mass of XK, XK/CuS0.5, XK/CuS1, and XK/CuS2 hydrogels were soaked in PBS for different days. As shown in Additional file 1: Fig. S1, all the hydrogels still maintained their original state with time, without fragmentation or dissolution. Moreover, as shown in Additional file 1: Fig. S2, after 7 days of soaking in PBS, the mass of all hydrogel groups did not decrease below 90%, which was sufficient to ensure the stability of the hydrogel on the wound during actual use.

### Biocompatibility of XK/CuS NPs hydrogel

**Fig. 6:**
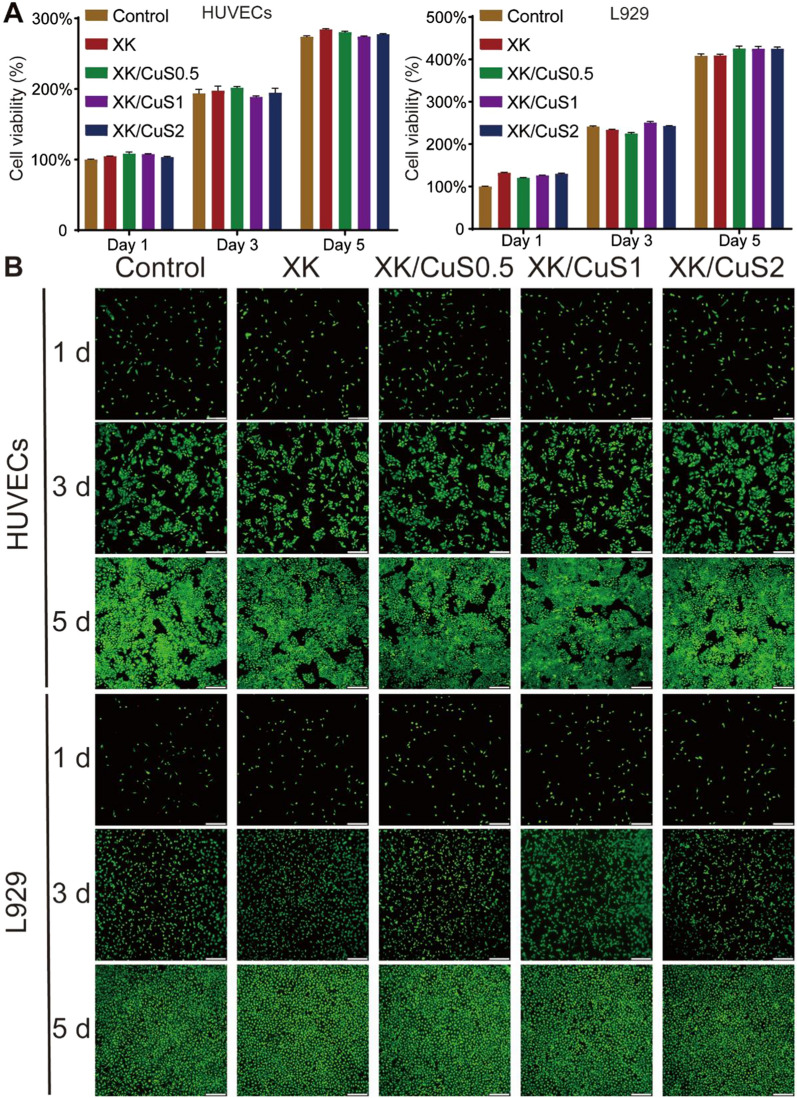
**A** Cell viability of HUVECs and L929 cells incubated with PBS (control), XK/CuS0, XK/CuS0.5, XK/CuS1, and XK/CuS2 hydrogels leachates, respectively. **B** The Live/Dead staining images of HUVECs and L929 cells after incubated with the PBS (control), XK, XK/CuS0.5, XK/CuS1, and XK/CuS2 hydrogels leachates for 1, 3, and 5 days, respectively. (Live cells: green, dead cells: red, scale bar: 200 μm)

Wound dressings should be in direct contact with the tissue and require favorable biocompatibility [54]. HUVECs and L929 cells were cultured with the leaching solutions of XK/CuS hydrogels to test their biocompatibility. The effect of the hydrogels on cell proliferation was tested by CCK-8 assay. As Fig. 6A exhibited, in comparison to the control group, the viability of HUVECs and L929 cells of XK/CuS groups exhibited the same rising trend on the 1st, 3rd, and 5th day, demonstrating that the XK/CuS hydrogel did not inhibit cell proliferation. Live/Dead staining of cells on days 1, 3, and 5 revealed that the majority of HUVECs and L929 cells were alive, while only a few dead cells were present (Fig. 6B). Also, the number of cells gradually grew over time, consistent with the CCK-8 results. These results suggested that XK/CuS hydrogels are harmless to cells and have excellent biocompatibility.

**Fig. 7:**
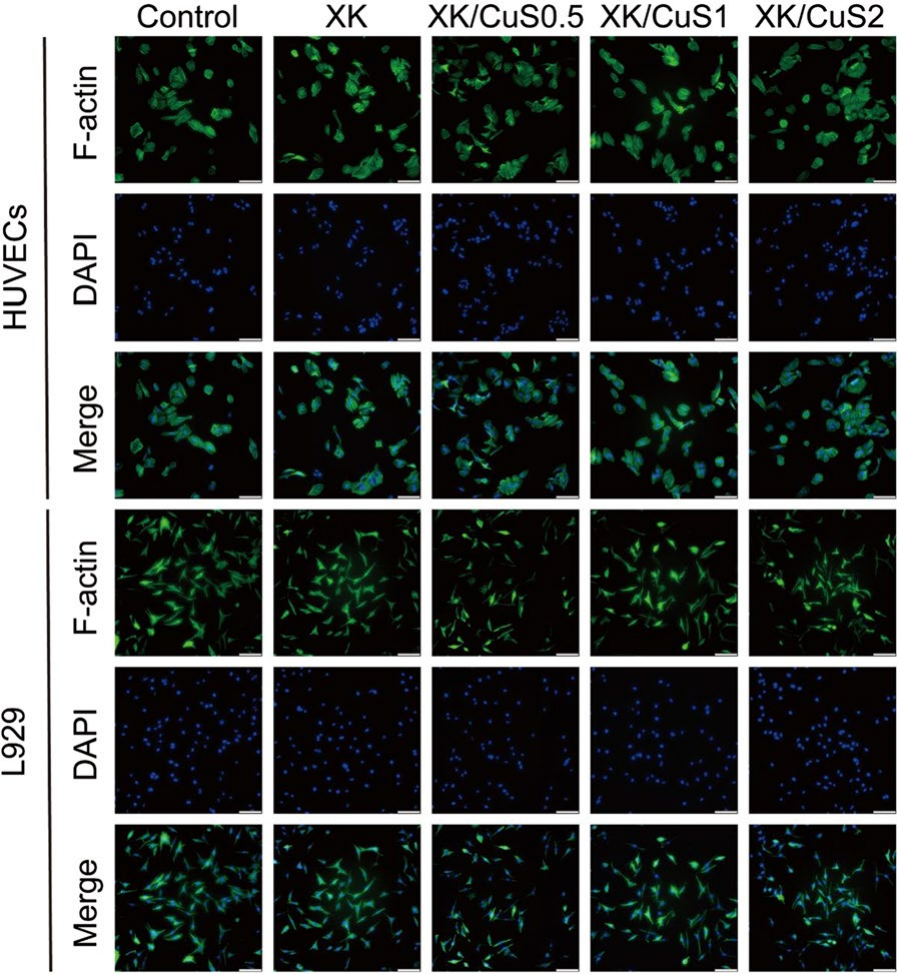
Fluorescent images of HUVECs and L929 cells co-cultured with PBS (control) and different leachates for 3 days, F-actin stained as green and nuclei stained as blue (scale bar: 100 μm)

The cells were co-cultured with hydrogel leachates for 3 days, and then cell morphology was observed. F-actin is one of the most important structural components of the cytoskeleton and is closely related to the morphology and structure of the cells [55]. The F-actin was stained with FITC-Phalloidin (green), and the nucleus was stained with DAPI (blue). As shown in Fig. 7, the HUVECs in the control and XK/CuS groups were polygonal with spreading cell morphology. The L929 cells in XK/CuS groups had the same visible filamentous foot as the control group, as well as showing similar numbers and distribution of filamentous F-actin. The morphological observation of cellular F-actin in XK/CuS showed that cells could grow normally in co-incubation with the leachates of XK/CuS hydrogels, which verified the excellent biocompatibility of XK/CuS hydrogels from another perspective.

### In vitro photothermal property of XK/CuS NPs hydrogel

**Fig. 8:**
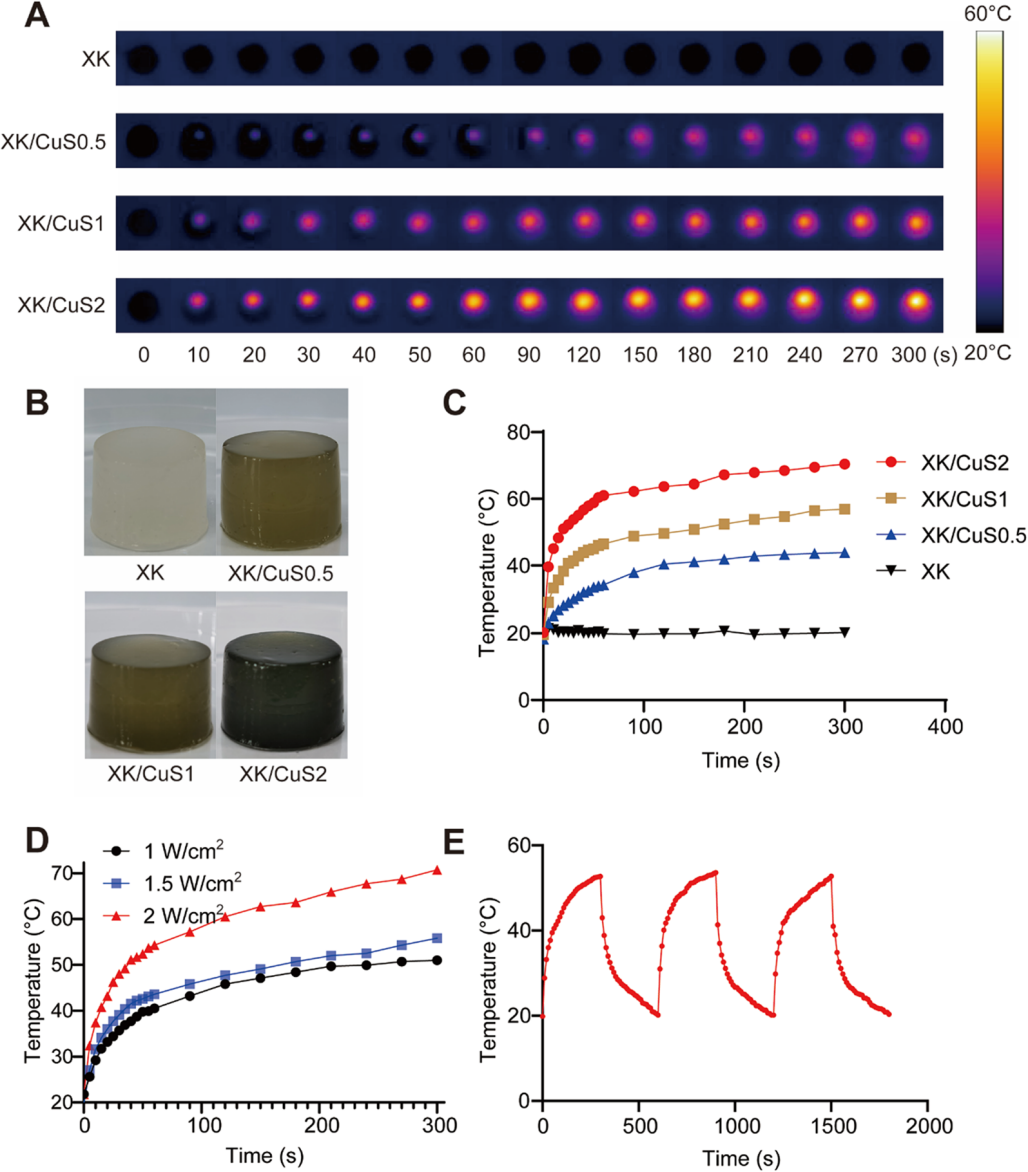
Photothermal property of XK/CuS hydrogels. **A** Infrared images of XK and XK/CuS hydrogels under 808 nm laser (1.5 W/cm^2^). **B** Photograph of XK and XK/CuS hydrogels. **C** Temperature profiles of XK and XK/CuS hydrogels under 808 nm laser (1.5 W/cm^2^). **D** Temperature profiles of XK/CuS1 under a laser power density of 0.5 W/cm^2^, 1.0 W/cm^2^, and 1.5 W/cm^2^. **E** The temperature profile of XK/CuS1 for 3 on–off cycles under 808 nm laser (1.5 W/cm^2^)

The photothermal property of XK/CuS NPs hydrogels was verified. The temperature of XK/CuS NPs hydrogels increased as the irradiation time became longer. XK hydrogel is white and has no photothermal effect. XK/CuS hydrogels are brown, and the higher the CuS NPs concentration, the darker the color and the stronger the photothermal effect (Fig. 8A, B). The CuS NPs concentrations of the hydrogels were screened, and the results are shown in Fig. 8C. The temperatures of the XK/CuS hydrogels all reached stability at 300 s. The temperature of XK/CuS0 did not change under 808 nm laser irradiation (1.5 W/cm^2^). The temperatures of XK/CuS0.5, 1, and 2 could reach up to 43 °C, 56 °C, and 70 °C, respectively, which were positively correlated with the concentration of CuS NPs. Compared with XK/CuS2 and XK/CuS0.5, the platform temperature of XK/CuS1 did not cause severe burns to surrounding tissues and was sufficient to kill bacteria, so XK/CuS1 hydrogel was chosen as the antibacterial wound dressing for the subsequent experiments [42]. The irradiation power was further screened (Fig. 8D). The temperatures of XK/CuS1 at 1 W/cm^2^, 1.5 W/cm^2^, and 2 W/cm^2^ after irradiation for 300 s were 51 °C, 55.8 °C, and 70.7 °C, respectively. After comparison, the power setting of 1.5 W/cm^2^ was more suitable. XK/CuS1 hydrogel could be warmed up to the previous temperature each time in the three on–off cycles under an 808 nm laser, showing that it also had photothermal stability (Fig. 8E).

### Antibacterial capacity of XK/CuS NPs hydrogel

**Fig. 9:**
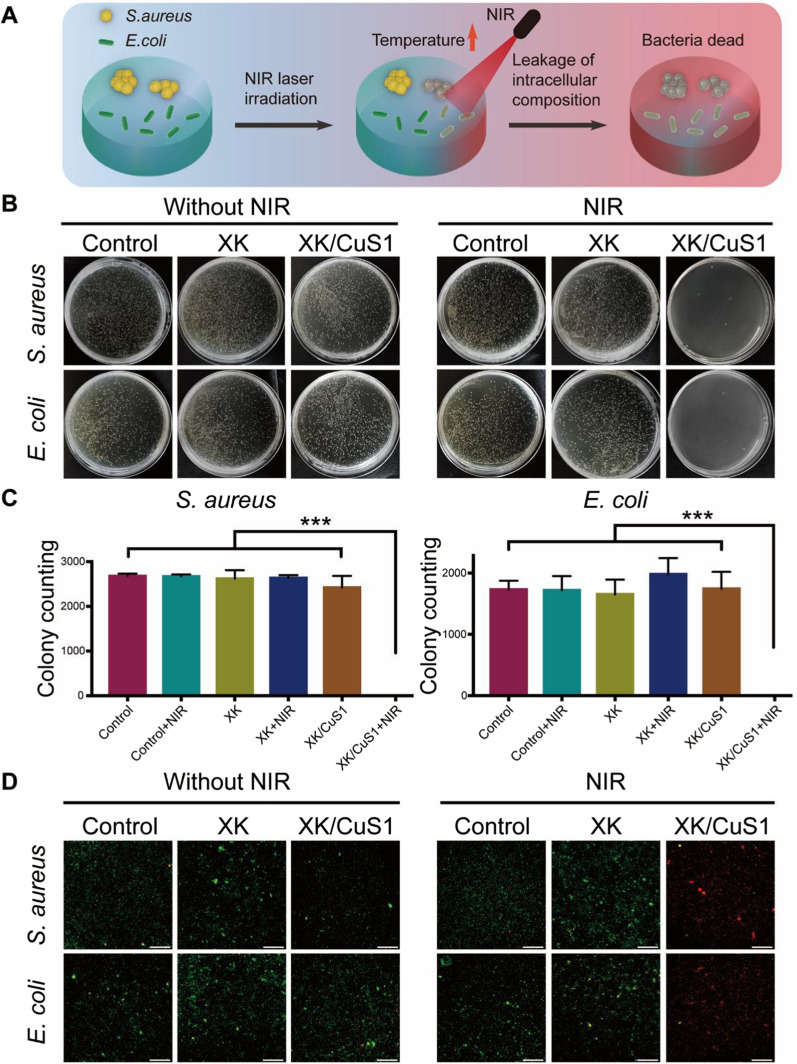
In vitro antibacterial activity of XK and XK/CuS1 hydrogels. **A** Schematic illustration of the antibacterial mechanism of XK/CuS hydrogels. **B** Representative colony formation of *S. aureus* and *E. coli* on LB agar plates treated with PBS, XK, and XK/CuS1 hydrogels with or without NIR irradiation (808 nm, 1.5 W/cm^2^) for 5 min, respectively. **C** The corresponding statistical data of bacterial colony counting of *S. aureus* and *E. coli*. Data were expressed as mean ± SDs, ***p < 0.001. **D** Live/dead bacteria staining photographs of *S. aureus* and *E. coli*. (Live bacteria: green, dead bacteria: red, scale bar: 100 μm)

Antibacterial capacity is one of the most important factors in assessing the function of a wound dressing [56]. Therefore, we analyzed the photothermal antibacterial ability of XK/CuS hydrogels. A schematic illustration of the antibacterial mechanism of XK/CuS hydrogels is shown in Fig. 9A. The mechanism of its antibacterial effect is the irreversible denaturation of bacterial proteins caused by high temperature, leading to the complete elimination of bacteria [57]. To research the effect of XK/CuS hydrogel on bacteria, the antibacterial properties of hydrogel were measured with the spread plate method and live/dead staining. It was evident from the number of colonies that the XK group had no antibacterial effect with or without NIR laser irradiation compared to the control group. XK/CuS1 group with NIR laser irradiation was almost no colony growth of either *S. aureus* or *E. coli*, while XK/CuS1 group without NIR laser irradiation had no antibacterial effect, illustrating that XK/CuS1 hydrogel has strong antibacterial performance under 808 nm laser irradiation (Fig. 9B). The results of bacterial colony counts were consistent with the colony growth (Fig. 9C).

The treated bacterial suspensions were centrifuged to collect the precipitates and dyed with a Live/Dead BacLight Bacterial Viability Kit. As shown in Fig. 9D, the bacteria in the control group, control + NIR group, XK group, XK + NIR group, and XK/CuS1 group mostly showed green fluorescence, suggesting that the bacteria were alive. The XK/CuS1 + NIR group appeared to have a large amount of red fluorescence, indicating that bacteria are almost killed, which also supported the above conclusion. These results suggest that XK/CuS1 hydrogel demonstrates awesome antibacterial performance ascribed to the photothermal property of CuS NPs.

### In vivo wound healing

**Fig. 10:**
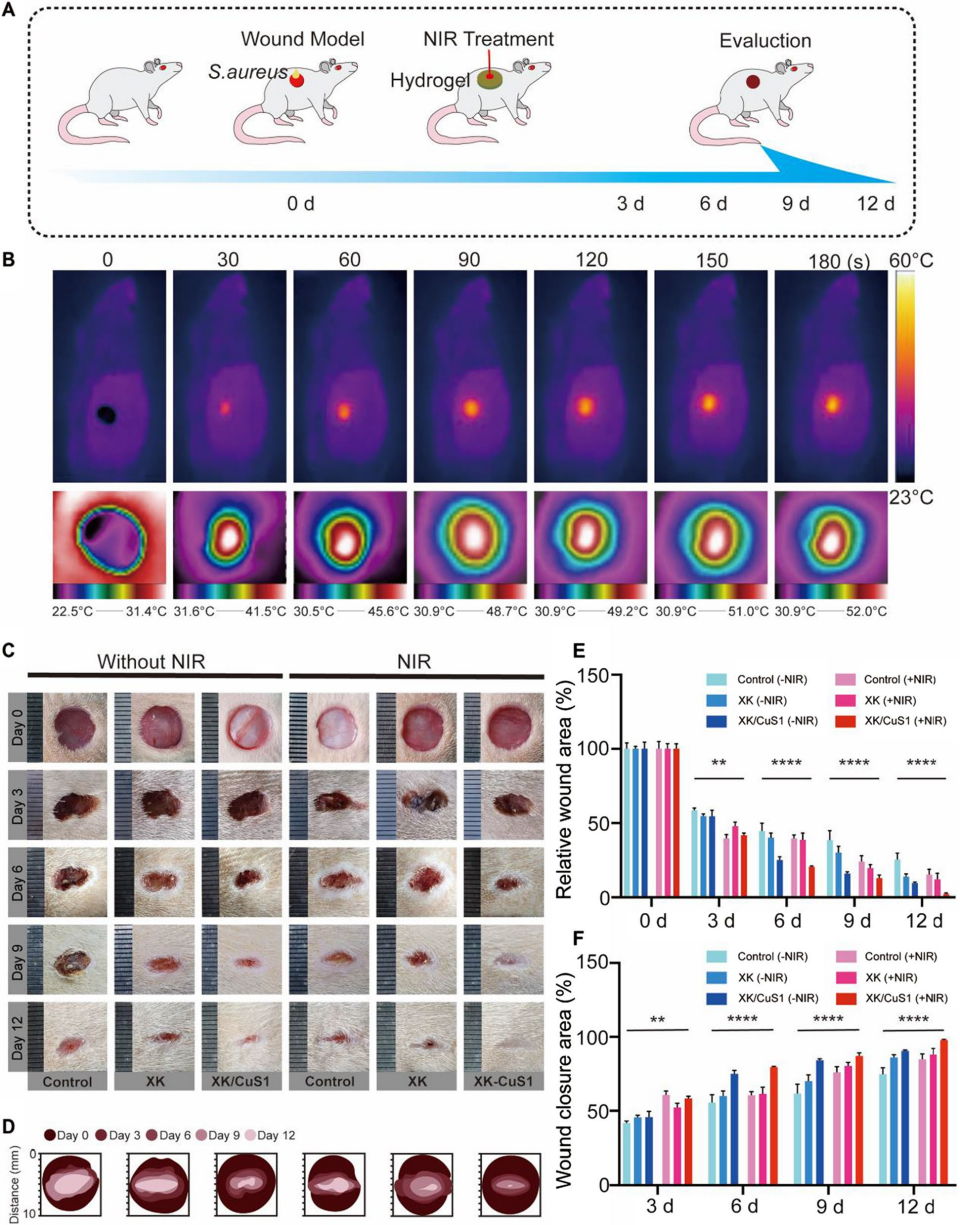
The ability of XK/CuS1 hydrogel to promote wound healing in vivo. **A** Schematic illustration of establishing a model of *S. aureus* infected rats and the process of hydrogel treatment. **B** Infrared images of the wound in the XK/CuS1 hydrogel + NIR group. **C** Representative data of wounds under *S. aureus* infection at days 0, 3, 6, 9, and 12. **D** The schematic diagram of the wound healing process. **E** Quantification of relative wound area at days 0, 3, 6, 9, and 12 for all groups. **F** Quantification of wound closure area on days 3, 6, 9, and 12 for all groups

The previous experiments showed that XK/CuS1 NPs hydrogel has good mechanical properties, biocompatibility, and photothermal antibacterial ability. Next, a full-skin wound infection model in SD rats was set up to further demonstrate the therapeutic effect of XK/CuS1 hydrogel on infected wounds at the animal level. XK/CuS1 hydrogel and XK hydrogel were placed on infected wounds, and the control group dealt with PBS on the infected wounds. All of them were divided into two groups with or without 808 nm laser irradiation, respectively. Wound healing of rats was observed on days 0, 3, 6, 9, and 12, and the experimental procedure is shown in Fig. 10A. After the 808 nm NIR laser treatment (1.5 W/cm^2^), the temperature change was monitored in real-time using the infrared thermographic camera. As shown in Fig. 10B, the temperature in the wound area reached stability after 2 min and was maintained at approximately 50 °C. This temperature is sufficient to kill bacteria without causing tissue damage, which is consistent with the consequences of in vitro assays [42].

The effect of different groups on wound repair was assessed by measuring the change in wound area at 0, 3, 6, 9, and 12 days. As shown in Fig. 10C, there was no significant difference between the XK group and the control group with or without NIR laser irradiation. The wounds in the XK/CuS1 group and XK/CuS1 + NIR group healed faster than the other groups. Moreover, the XK/CuS1 + NIR group healed more rapidly and was completely healed on the 12th day. The wound area at each time point was quantified and statistically analyzed (Fig. 10D–F), and the results showed that there was a significant difference in the capability of accelerating wound healing in the XK/CuS1 + NIR group compared to the control group. This is mainly ascribed to the high temperature generated by CuS NPs under NIR laser irradiation could eliminate the bacteria in the wound and reduce the inflammatory response, thus decreasing the tissue damage by immunocytes and accelerating the wound healing [6].

### In vivo histopathological analysis

**Fig. 11:**
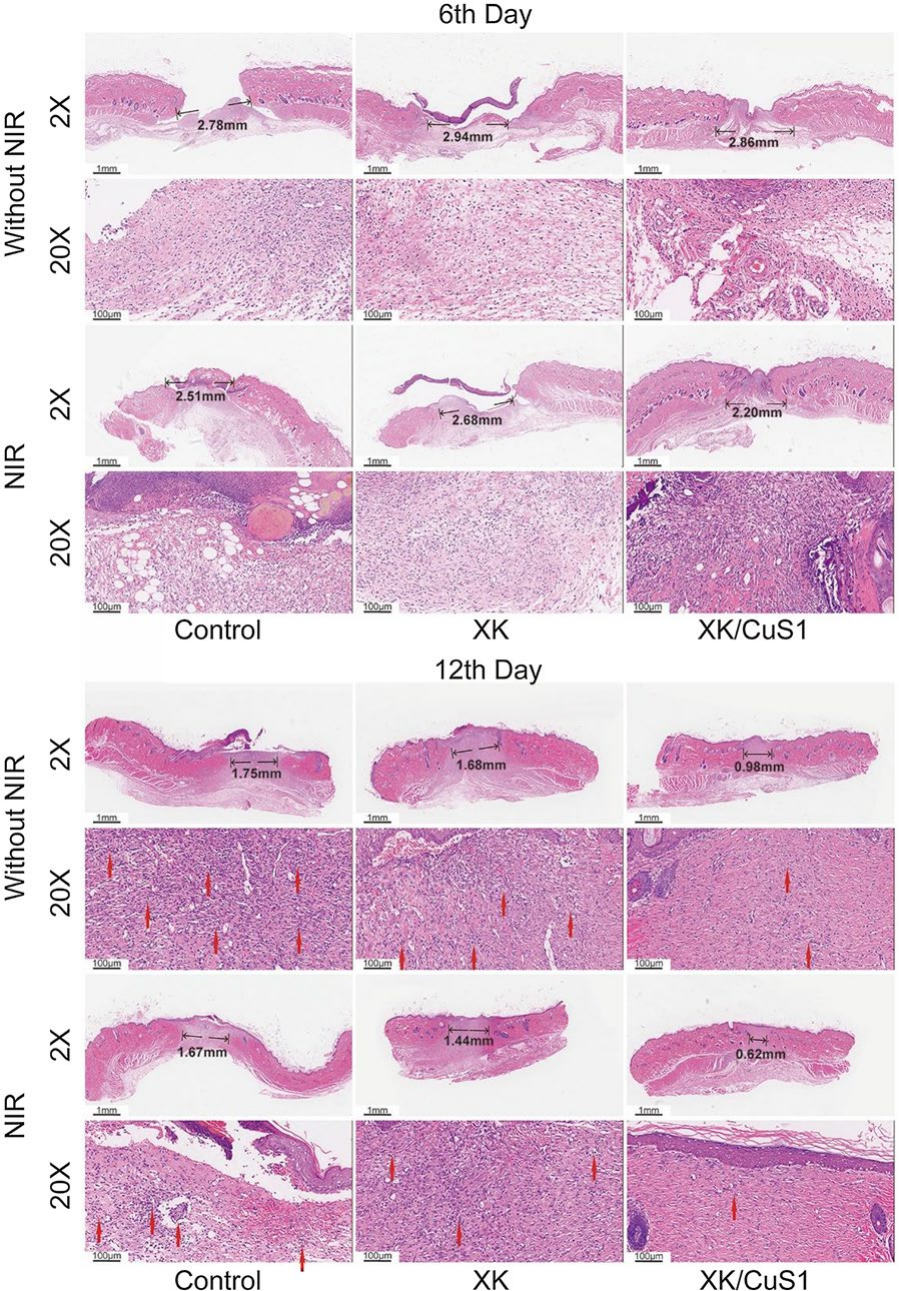
H&E staining of the full-skin wound on days 6 and 12. Red arrows indicated inflammatory cells; black arrows indicated epithelial space

The effect of XK/CuS NPs hydrogel on wound healing was further evaluated by histological analysis. H&E staining is shown in Fig. 11. On the 6th day, the epithelial space of the XK/CuS1 + NIR group was 2.20 mm, while the other groups were more than 2.50 mm. The wound tissue of the XK/CuS1 + NIR group grew faster than the other groups and formed epithelial tissue.

On the 12th day, inflammation cells were still present in the tissues of the control and XK hydrogel groups (red arrows), indicating that there was still an inflammatory response, while the XK/CuS1 + NIR group was almost devoid of inflammatory cells. It illustrates that XK/CuS NPs hydrogel can remove bacteria under 808 nm laser radiation and reduce the duration of the inflammatory response. In addition, the epithelial space of the XK/CuS1 + NIR group was narrower than others (black arrows), indicating that the XK/CuS1 + NIR group had the best wound recovery.

**Fig. 12:**
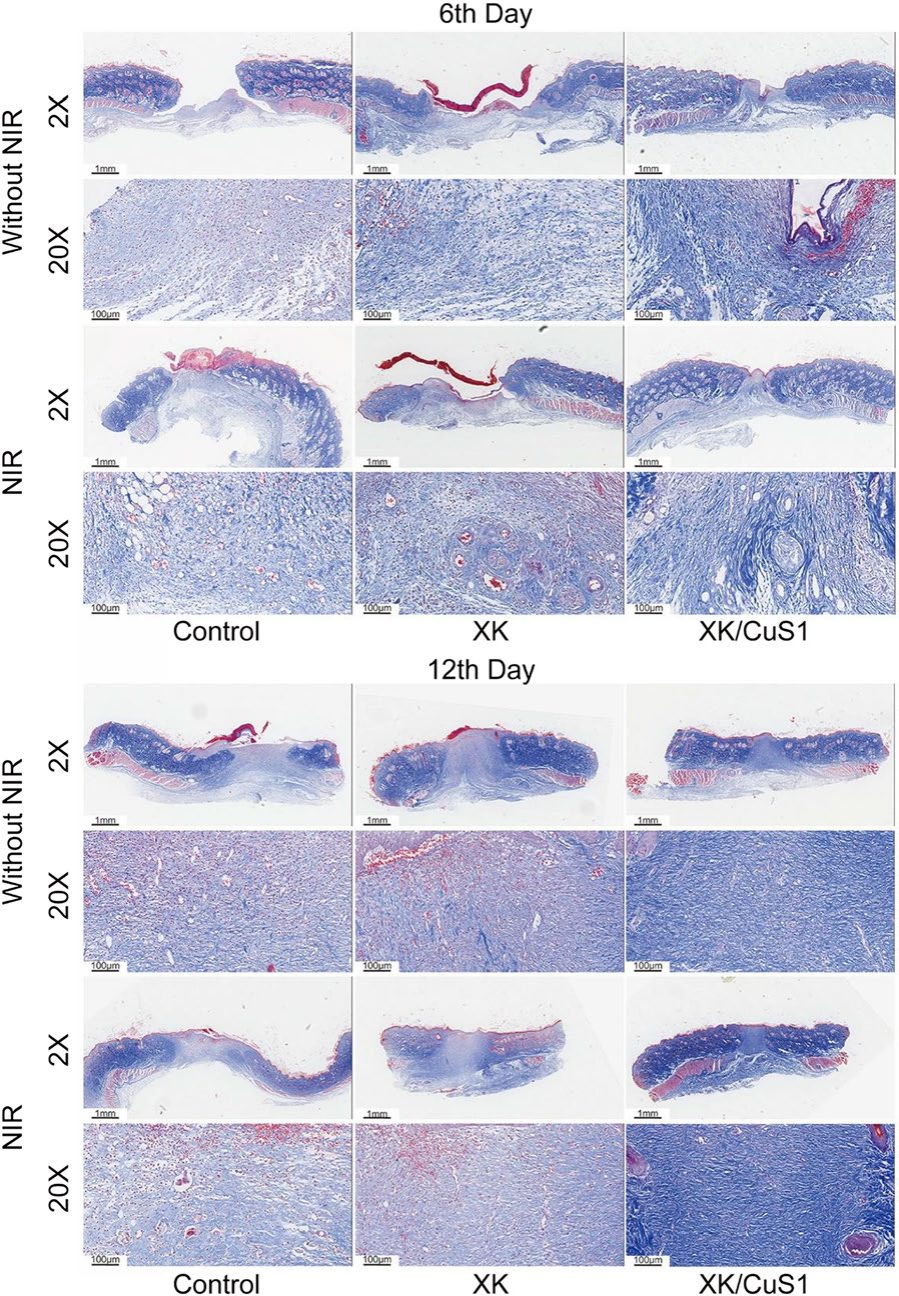
Masson’s trichrome staining of the full-skin wound on days 6 and 12. The collagen fibers were dyed blue. Mature collagen fibers are arranged in lines and kept densified

As a major component of the extracellular matrix, collagen not only plays a role in physical support but also plays a very important part in cell activities, including cell multiplication, migration, and differentiation, and is an important indicator of wound healing [58]. The collagen fibers were observed by Masson staining. As shown in Fig. 12, the staining of the XK/CuS1 + NIR group on the 6th and 12th day was deeper than other groups, reflecting more collagen deposition, while the rest of the groups had very light staining. In addition, the collagen in the XK/CuS1 + NIR group on the 12th day was orderly, demonstrating that the XK/CuS1 + NIR group could effectively promote the deposition of collagen fibers, thus facilitating wound healing.

**Fig. 13:**
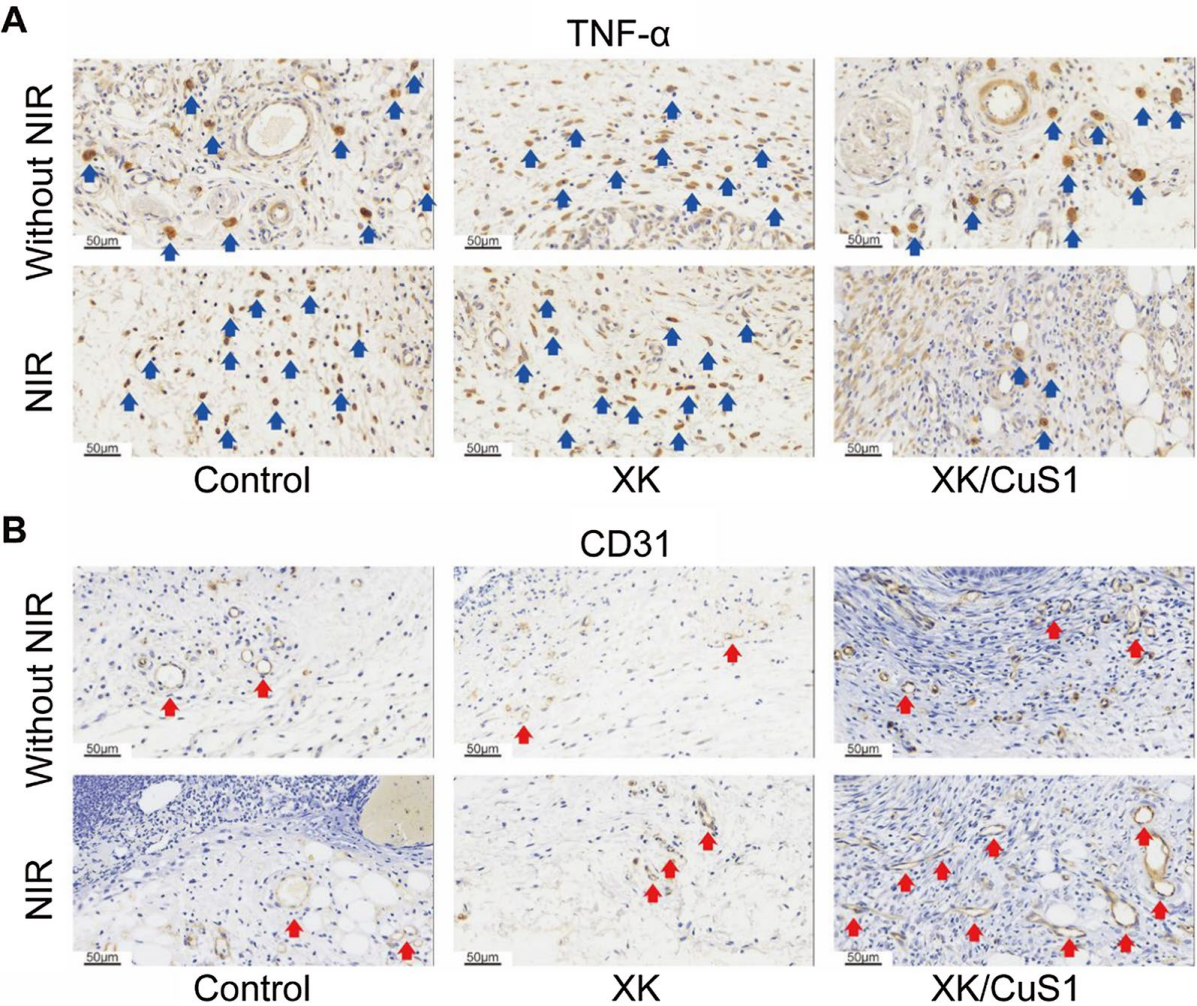
**A** TNF-α and **B** CD31 immunohistochemical images at day 6. Blue arrow: TNF-α positive cells; red arrow: blood vessels

During wound healing, the persistent inflammatory response leads to local tissue cell necrosis and delays the wound healing process [59]. To assess the early inflammation level, the wound tissue section on the 6th day was immunohistochemically stained for TNF-α [60]. As shown in Fig. 13A, significant TNF-α expression was found in the control and XK groups with or without NIR laser irradiation. However, the TNF-α expression level in the XK/CuS1 + NIR group was lower than the others, suggesting that the XK/CuS1 + NIR group can bring down inflammation.

Angiogenesis is important for wound healing; neovascularization provides oxygen, growth factors, and immune support to the healing tissue [61]. CD31 is usually highly expressed on the surface of vascular endothelial cells and serves as a marker of angiogenesis [62]. Therefore, CD31 immunohistochemical staining was performed on the wound slices on the 6th day. As shown in Fig. 13B, high CD31 expression was observed in the XK/CuS1 + NIR group compared with the rest of the groups, indicating that vascular angiogenesis of the XK/CuS1 + NIR group was superior to the others. All the results suggest that XK/CuS1 hydrogels with NIR laser irradiation reduce the inflammatory response, promote angiogenesis, and thus accelerate wound healing.

## Conclusion

In general, for the first time, we ingeniously synthesized CuS NPs in situ using natural sericin as bio-templates. The CuS NPs obtained by this method are evenly sized and have favorable photothermal properties. Subsequently, CuS@Ser NPs were loaded into xanthan/konjac glucomannan hydrogel as a photothermal agent, and the obtained XK/CuS NPs hydrogel has outstanding mechanical properties, which compression stress higher than 900 kPa at 80% compression strain, as well as excellent compressive stability and mechanical flexibility. Most importantly, benefiting from the good photothermal properties of CuS@Ser NPs, the XK/CuS NPs hydrogel has a stable photothermal antibacterial capacity to eradicate infected wound bacteria. The mechanism of the antibacterial effect is attributed to the irreversible denaturation of bacterial proteins induced by high temperature. The full-skin wound infection model in SD rats further confirmed that XK/CuS hydrogels could accelerate wound healing under NIR irradiation by providing anti-infection capability, reducing inflammatory reactions, and promoting angiogenesis. All these results indicate that XK/CuS NPs hydrogel has excellent mechanical and antimicrobial properties that could accelerate the healing of infected wounds. This work also provides an innovative strategy for designing photothermal antimicrobial wound dressings.

### Supplementary Information

**Additional file 1: Figure S1.** Photograph of XK, XK/CuS0.5, XK/CuS1, and XK/CuS2 hydrogel after soaking in PBS for 0, 1, 3, 5, and 7 days, respectively. **Figure S2.** Weight change of XK, XK/CuS0.5, XK/CuS1, and XK/CuS2 hydrogel after soaking in PBS for 0, 1, 3, 5, and 7 days, respectively.

## Data Availability

The data that support the findings of this study are available from the corresponding author upon reasonable request.
