# Supplementary material for: Facile and eco-friendly fabrication of biocompatible hydrogel containing CuS@Ser NPs with mechanical flexibility and photothermal antibacterial activity to promote infected wound healing
Source: J Nanobiotechnology. 2023 Aug 10;21:266. doi: 10.1186/s12951-023-02035-6 (PMC10416498; doi:10.1186/s12951-023-02035-6)
Supplement: Supplementary file 1 — Additional file 1: Figure S1. Photograph of XK, XK/CuS0.5, XK/CuS1, and XK/CuS2 hydrogel after soaking in PBS for 0, 1, 3, 5, and 7 days, respectively. Figure S2. Weight change of XK, XK/CuS0.5, XK/CuS1, and XK/CuS2 hydrogel after soaking in PBS for 0, 1, 3, 5, and 7 days, respectively. [file 12951_2023_2035_MOESM1_ESM.docx]

**Supporting Information**

**Facile and eco-friendly fabrication of biocompatible hydrogel containing CuS@Ser NPs with mechanical flexibility and photothermal antibacterial activity to promote infected wound healing**

Ye Guo, Bingqing Xie, Min Jiang, Lingling Yuan, Xueyu Jiang, Silei Li, Rui Cai, , Junliang Chen, Xia Jiang, Yun He*, Gang Tao*


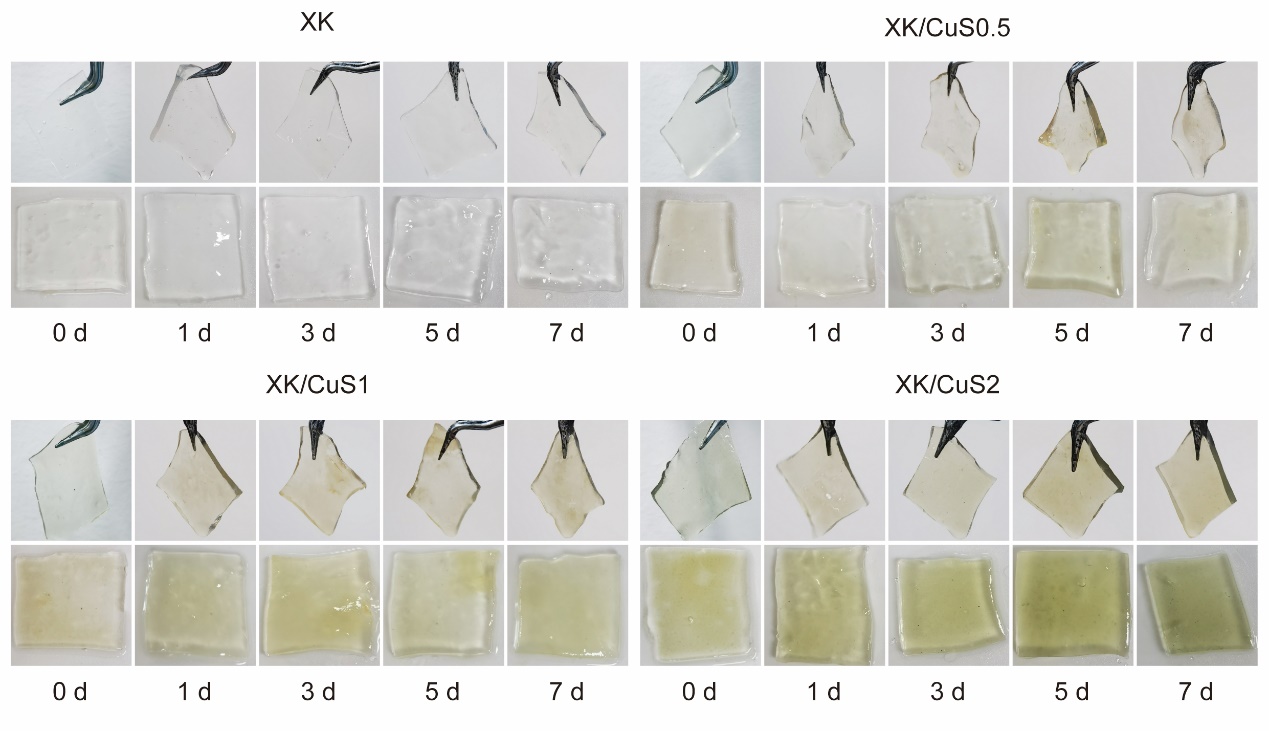


Fig. S1. Photograph of XK, XK/CuS0.5, XK/CuS1, and XK/CuS2 hydrogel after soaking in PBS for 0, 1, 3, 5, and 7 days, respectively.


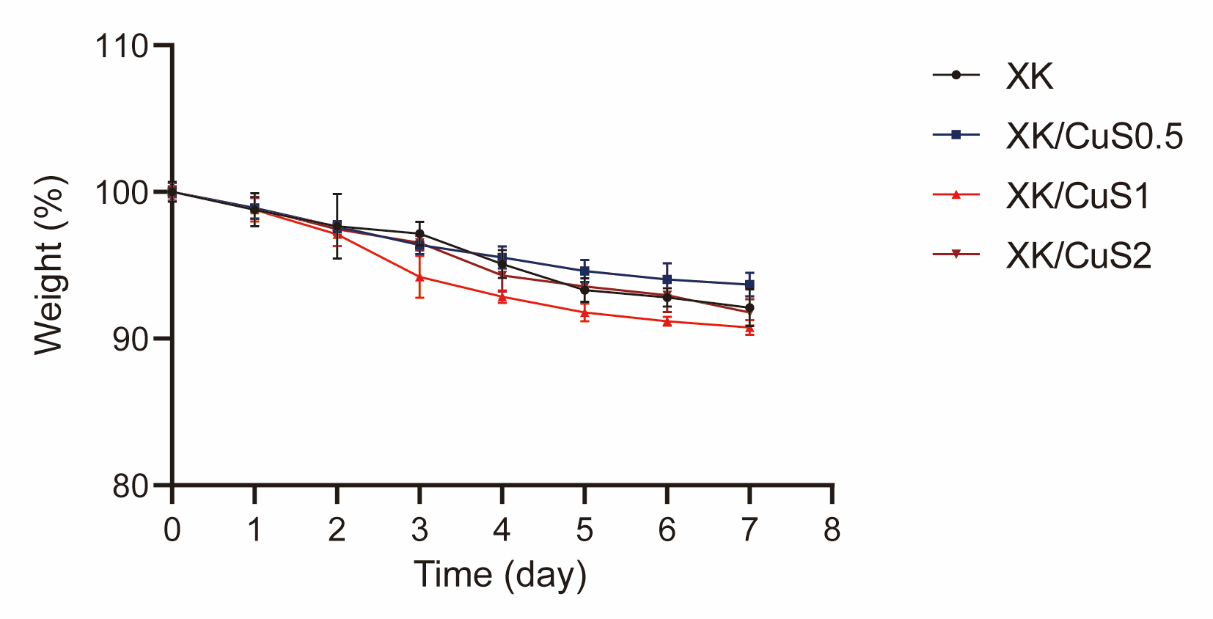


Fig. S2. Weight change of XK, XK/CuS0.5, XK/CuS1, and XK/CuS2 hydrogel after soaking in PBS for 0, 1, 3, 5, and 7 days, respectively.
